# Effects of semicarbazide-sensitive amine oxidase inhibitors on morphology of aorta and kidney in diabetic rats

**DOI:** 10.1186/s12902-019-0392-1

**Published:** 2019-06-10

**Authors:** Chaosheng Li, Zhenhua Wang, Xiaoli Li, Jun Chen

**Affiliations:** 1 Department of Cardiology, People’s Hospital of Baoan District, No.118 Longjing second road, Baoan District, Shenzhen, 518100 China; 2 Shenzhen Hospital of Southern Medical University, Baoan District, Shenzhen, 518100 China

**Keywords:** Aminoguanidine, 2-bromoethylamine, Semicarbazide-sensitive amine oxidase, Formaldehyde, Vascular dysfunction

## Abstract

**Background:**

The present study aimed to investigate the inhibitory effects of aminoguanidine (AG) and 2-bromoethylamine (2-BEA) on the semicarbazide-sensitive amine oxidase (SSAO) activity both in vitro and in vivo, and the prevention role of AG and 2-BEA in the morphology of aorta and kidney in diabetic rats.

**Methods:**

The aortic homogenates isolated from Sprague-Dawley (SD) rats were treated with different concentrations of AG or 2-BEA to investigate the inhibitory effects on the SSAO activity in vitro, using benzylamine as the substrate. In addition, 65 male SD rats were randomly assigned into normal control (NC) (*n* = 10), NC + AG (*n* = 10), NC + 2-BEA (*n* = 10) and diabetes mellitus (DM) model groups (*n* = 35). Type 1 diabetic rat model was induced by intraperitoneal injection of 1% streptozotocin-sodium citrate buffer 55 mg/kg. After establishing the diabetic rat model by a single intraperitoneal injection of streptozotocin. Except those failed in modeling, 30 rats in the DM model group were further randomly divided into the DM, DM + AG, DM + 2-BEA groups (*n* = 10 in each). Rats in the DM + AG and NC + AG group were intraperitoneally injected with AG (25 mg/kg),those in the DM + 2-BEA and NC + 2-BEA group were administered with 2-BEA (20 mg/kg) daily for eight weeks. After eight weeks of treatment, the SSAO activity in the plasma and aorta, and plasma levels of formaldehyde (FA) and methylamine (MA) were measured by high performance liquid chromatograph. Radioimmunoassay was used to determine the plasma endothelin-1 (ET-1) concentration, while nitric acid deoxidized enzyme method was performed to detect the plasma nitrate/nitrite (NO(x)-) level. Besides, the morphological changes of aorta and kidney tissues were examined by optical and electron microscopes.

**Results:**

Both AG and 2-BEA exerted strong inhibitory effect on the aortic SSAO activity in vitro, with the IC_50_ values of 12.76 μmol/L and 3.83 μmol/L, respectively. Compared with the NC group, the SSAO activity in the plasma and aorta, and plasma levels of MA and ET-1 were significantly increased (*P* < 0.01), whereas the plasma NO(x)- level was obviously lower in the DM group (*P* < 0.01). A significantly decreased SSAO activity and plasma ET-1 level, as well as obviously increased plasma levels of MA and NO(x)- were observed in the DM + AG and DM + 2-BEA groups in comparison with the DM group (*P* < 0.01). However, there was no significant difference in plasma FA concentration among all the groups. Besides, the morphological changes of aorta and kidney were apparently alleviated in the DM + AG and DM + 2-BEA groups as compared with the DM group.

**Conclusions:**

Both AG and 2-BEA can inhibit the SSAO activity in the plasma and aorta. Moreover, the inhibitory effects of AG and 2-BEA on the SSAO-mediated oxidative deamination had great benefit in the morphological changes of aorta and kidney in diabetic rats.

## Background

It has been found that vascular dysfunction in diabetes mellitus (DM) have a direct impact on the prognosis of DM and quality of life, and they have been proven to contribute to the high mortality and disability in patients suffering from DM. Thus, the prevention of vascular dysfunction is of vital importance in the treatment of DM. Diabetic vascular dysfunction can develop into systemic vascular damage, which is mainly divided into macrovascular disease(aortic atherosclerosis, coronary artery disease, increased risk of cerebrovascular disease, peripheral vascular disease), microangiopathy(diabetic nephropathy, diabetic retina, Lesions, diabetic neuropathy, diabetic cardiomyopathy). Semicarbazide-sensitive amine oxidase (SSAO) has been shown to catalyze the deamination of endogenous methylamine (MA) and aminoacetone, resulting in the production of toxic formaldehyde (FA), hydrogen peroxide (H_2_O_2_), and ammonia which were damage to endothelial cells. Previous published studies have shown that the activity of SSAO was greatly increased in the serum of patients with DM [1, 2], and the increased SSAO activity was reported to be positively correlated with the severity of DM complicated by retinopathy, nephropathy, and atherosclerosis [3, 4]. 2-bromoethylamine (2-BEA) and aminoguanidine (AG) can be viewed as a highly potent, selective, and suicide inhibitors of SSAO. A variety of researches have demonstrated AG was capable of preventing the occurrence and development of diabetic vascular dysfunction by inhibition of the production of advanced glycated end products (AGEs) [5, 6]. The prevention of diabetic vascular dysfunction by SSAO inhibitors was rarely reported at home and abroad. Therefore, the aim of the present study was to investigate the effects of AG and 2-BEA on the SSAO activity in vitro and in vivo and to evaluate whether the inhibition of SSAO activity had a beneficial effect on the morphological changes of aorta and kidney in diabetic rats. The mechanisms of SSAO-mediated oxidative deamination involved in the prevention of diabetic vascular dysfunction have also been explored in this study.

## Methods

### Animals

Male Sprague-Dawley rats (270-310 g) were purchased from the Guangdong Medical Laboratory Animal Center(license number: SYXK (Yue) 2008–0002). The animals were housed in hanging wire cages and kept under a standard laboratory condition (19–27 °C, relative humidity of 40–70%, ammonia concentration of ≤14 mg/m^3^ and noise levels of ≤60 dB) on a 12-h light/dark cycle. The rats were allowed free access to food and water.

### Preparation of working standard solutions

AG (11.055 mg; Sigma Chemical Co., St. Louis, Mo, USA) was dissolved in 10 mL of phosphate-buffered saline (PBS, 0.1 mol/L, pH 7.4) to make a concentration of 10 mmol/L. This standard solution was further diluted with PBS to final concentrations of 4000, 2000, 1000, 500, 200, 100, 50, 10, and 2 μmol/L. Meanwhile, 2-BEA (20.489 mg; Sigma Chemical Co., St. Louis, Mo, USA) was also prepared in 10 mL of PBS to a concentration of 10 mmol/L. After further dilution, the final concentrations of working standard solutions of 2-BEA were 1000, 500, 200, 160, 100, 80, 40, 20, and 10 μmol/L.

### Preparation of aortic homogenates

The rats were sacrificed by cervical dislocation, and the aorta was rapidly dissected, washed with ice-cold PBS, dried on filter paper, and then weighed. After the addition of PBS, the aortic samples were cut into pieces and homogenized using a high-speed homogenizer (Ultra-Turrax, IKA® Werke, Staufen, Germany) at 22000 rpm. Subsequently, the homogenates were centrifugated at 1000 rpm for 10 min at 4 °C. The supernatants were separated and stored until assayed for the SSAO activity in the aorta.

### Enzymatic reaction in vitro

One hundred μL of the supernatants of aortic tissue homogenates were collected into 1.5-mL centrifuge tubes. Then, 20 μL a series of different concentrations of AG or 2-BEA working standard solutions, 20 μL of clorgyline (Sigma Chemical Co., St. Louis, Mo, USA), and 60 μL of PBS were added into each tube. Meanwhile, negative controls were established by replacing the drugs with PBS. After the addition of 200 μL of benzylamine (Sigma Chemical Co., St. Louis, Mo, USA), the mixture was allowed to stand for 1 h in a water bath at 37 °C. The reaction was stopped by adding 100 μL of 20% trichloroacetic acid (Guangzhou Chemical Reagent Co., Ltd., Guangzhou, Guangdong, China). Next, 300 μL of the supernatants were collected by centrifugation into 5-mL centrifuge tubes, followed by the addition of 50 μL of dinitrophenylhydrazine (DNPH; Sigma Chemical Co., St. Louis, Mo, USA). The DNPH derivatives were extracted with ethyl acetate, and the organic layer was dried under a nitrogen stream at 45 °C. Finally, the resultant residue was dissolved in 500 μL of acetonitrile-0.1% formic acid, until being assayed by high performance liquid chromatography (HPLC) system (Agilent Technology 1200 series, Santa Clara, USA).

### Animal models and grouping

The rats were randomly assigned to normal control (NC) (*n* = 10), NC + AG (*n* = 10), NC + 2-BEA (*n* = 10) and DM model groups (*n* = 35). After a 12-h fasting, rats in the DM model group were administered with 1% streptozotocin (STZ) dissolved in sodium citrate buffer at a dose of 55 mg/kg by intraperitoneal injection, while rats in the NC, NC + AG, and NC + 2-BEA groups were intraperitoneally injectied with equal volumes of sodium citrate buffer. After one week, the blood was withdrawn by nicking the lateral tail vein for measurement of blood glucose level. The diabetic rat model was successfully established if the blood glucose level was of > 16.7 mmol/L for two consecutive measurements. Except those failed in modeling, 30 rats from the DM model group were selected, and they were randomly assigned to the DM, DM + AG, and DM + 2-BEA groups, with 10 rats in each group. Rats in the NC + AG and DM + AG groups were intraperitoneally injected with AG at a daily dose of 25 mg/kg for eight weeks, while rats in the NC + 2-BEA and DM + 2-BEA groups were administered with 2-BEA at a daily dose of 20 mg/kg by intraperitoneal injection for eight weeks. Meanwhile, rats in the NC and DM groups were intraperitoneally injectied with deionized water at a daily dose of 0.5 mL/100 g.

### Sample collection

After eight weeks of treatment, rats in each group were anesthetized with an intraperitoneal injection of 1% pentobarbital sodium. Then, blood samples were drawn from the abdominal aorta, transferred into dried tubes containing EDTA as an anticoagulant, and centrifuged at 3000 rpm for 10 min at 4 °C. The supernatant liquid of blood was separated and stored at − 80 °C, until being assayed for the plasma SSAO activity, and levels of MA, FA, plasma nitrate/nitrite NO(x)-, and endothelin-1 (ET-1) in the plasma. Meanwhile, the aorta was quickly removed through a thoracotomy, select the 1.5 cm artery segment at the origin of aortic arch, cut the artery longitudinally with the ophthalmic scissors and attach the endothelium to the filter paper, rinse the normal asline and place in formalin solution. Fixation, routine dehydration, paraffin embedding, HE staining, analyzed by optical microscopy (Olympus, Tokyo, Japan). Afterward, cuted approximately 1 cm long aorta into 4% glutaraldehyde phosphate buffer (pH 7.4), were examined under a transmission electron microscope (Olympus, Tokyo, Japan). The other aortic segments were used for determining the SSAO activity. Additionally, the right kidney was quickly removed, fixed with 10% formaldehyde, and stained with H&E, while the cortex of left kidney was dissected out for subsequent examination by electron microscopy.

### Variables assessed

The tail vein of each rat was nicked with a fresh scalpel, and a glucose test strip immediately spotted with blood and analyzed using a glucose meter every two weeks throughout the treatment period. The plasma SSAO activity and levels of MA and FA were determined by HPLC [7, 8]. The analysis for plasma SSAO activity was carried out on an Agilent Zorbax SB C_18_ column (150 × 2.1 mm, 5 μm) with the detecting wavelength of 382 nm. The plasma level of MA was measured by HPLC equipped with fluorescence detection. This method was performed on a HP Zobrax Stable Bond SB C_18_ (150 × 4.6 mm, 5 μm) using a gradient elution procedure, with the excitation and emission wavelengths of 350 and 530 nm, respectively. Meanwhile, the FA level in the plasma was analyzed on an Agilent Zorbax SB C_18_ column (150 × 2.1 mm, 5 μm) and detected by a fluorescence detector, with the excitation and emission wavelengths of 346 and 442 nm, respectively. Additionally, the NO(x)- level was detected by nitric acid deoxidized enzyme method using the specific detection kit (Jiancheng Bioengineering Institute, Nanjing, Jiangsu, China). The nitrate nitrite (NO2-) and nitrate (NO3-) levels were measured by nitrate reductase colorimetric method to reflect the plasma NO(x)- level indirectly. Plasma NO2- is further converted to NO3-, this method uses nitrate reductase specific to reduce NO3- to NO2-, by measuring the depth of color can be used to calculate the level of NO(x)-, the operation process is strictly in accordance with the kit instructions, the colorimeter using a microplate reader, 540 nm, 96-well colorimetric plate to measure the absorbance of each hole, according to the standard curve calculate the NO(x)- content. The plasma ET-1 level was measured with a radioimmunoassay kit (Jiancheng Bioengineering Institute, Nanjing, Jiangsu, China).

### Morphological analysis

The aortic segments (1.5 cm in length) from its origin and the right kidney were fixed with 10% neutral buffered formalin, dewaxed with xylene, and embedded in paraffin. The paraffin-embedded sections were then routinely cut into 4-μm slices, stained with H&E, and analyzed by optical microscopy. In addition, the segments of aorta (1.0 cm in length) and cortex of the left kidney were rinsed with a fixative solution, and then transferred to a clear paraffin board at low temperatures. After removal of the cut surface, the samples were cut into 1 mm^3^ slices and fixed with 2.5% glutaraldehyde within 1 min. The samples were further fixed with 1% osmium tetroxide, dehydrated through an ethanol gradient, infiltrated with embedding medium, and cut into slices, followed by double-staining with sodium phosphate and lead citrate and detection under an electron microscopy.

### Data analyses

The half maximal inhibitory concentrations (IC_50_) for AG and 2-BEA were calculated using GraphPad Prism 6.0 software (GraphPad, San Diego, CA, USA), and statistical analyses were performed using SPSS version 19.0 statistical software (SPSS Inc., Chicago, IL, USA). Data were expressed as mean ± standard deviations (SD). One-way analysis of variance was used to compare the differences among groups. Values of *P* < 0.05 were considered statistically significant.

## Results

### Inhibitory effects of AG and 2-BEA on aortic SSAO activity

**Fig. 1:**
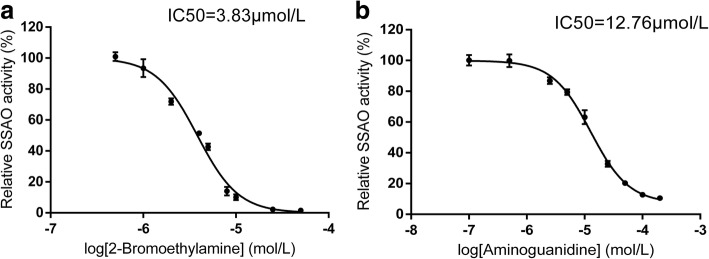
Inhibition of aortic SSAO activity exerted by AG and 2-BEA in vitro (*n* = 3). SSAO activity was measured by HPLC method using benzylamine as substrate. For assessment of inhibitory effect, SSAO was pre-incubated in the presence or absence of different concentrations of 2- BEA (**a**) or AG (**b**) before the addition of benzylamine. SSAO, semicarbazide-sensitive amine oxidase; AG, aminoguanidine; 2-BEA, 2-bromoethylamine

Both AG and 2-BEA exerted their inhibitory effect on the aortic SSAO activity in vitro, with the IC_50_ values of 12.76 μmol/L and 3.83 μmol/L, respectively (Fig. 1). Moreover, the inhibitory effect of 2-BEA on the aortic SSAO activity was approximately three times stronger than that of AG.

### Comparison of the body weight and blood glucose level

**Fig. 2:**
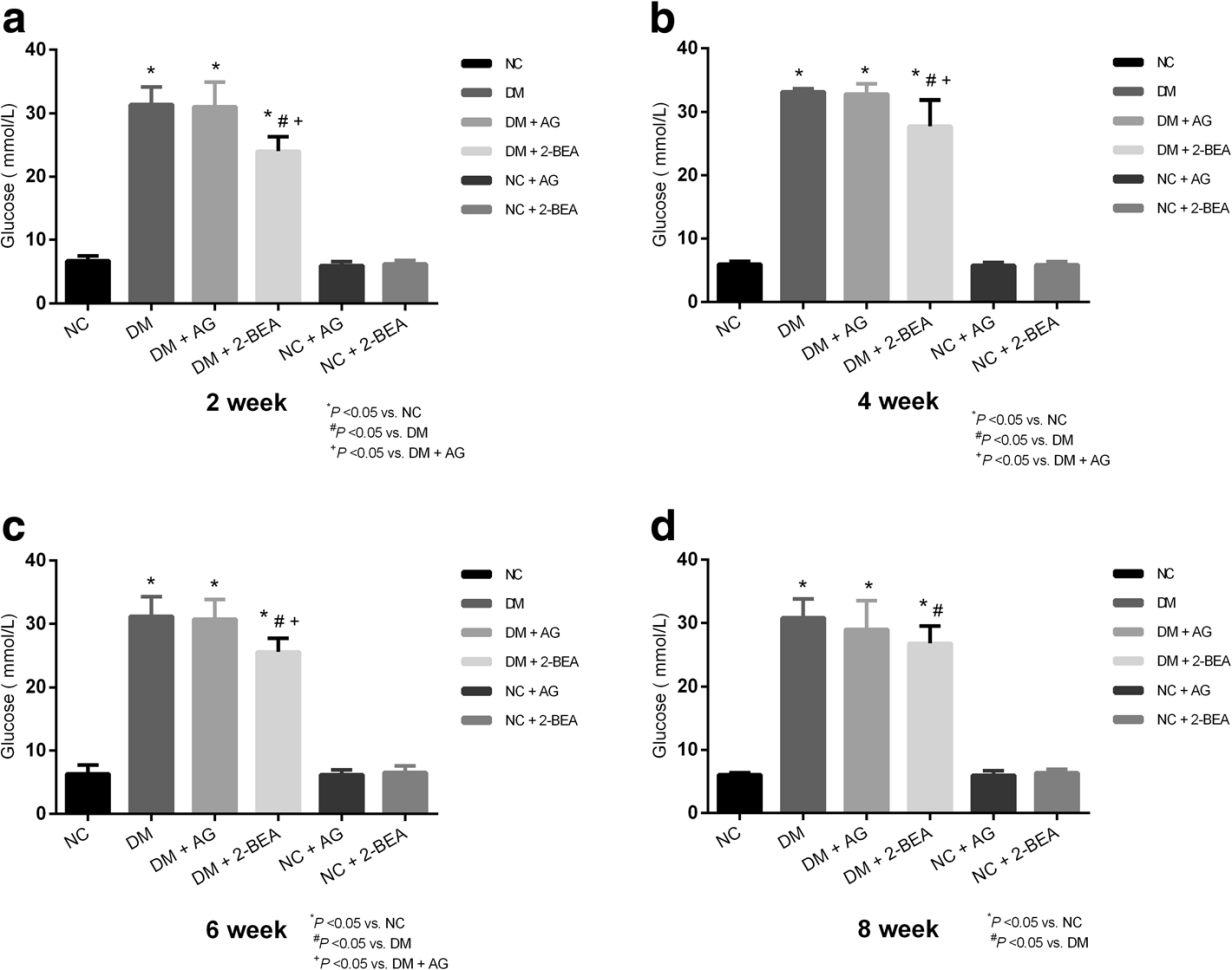
Comparison of blood glucose level in different groups of rats (*n* = 10). After establishing the diabetic rat model, the blood glucose levels among all the groups at respective time points of 2 (**a**), 4 (**b**), 6 (**c**), and 8 (**d**) week was measured. Results are reported at the mean ± SD. ^*^P < 0.05 *νs.* NC group, ^#^*P* < 0.05 vs. DM group, ^+^*P* < 0.05 vs. DM + AG group

**Fig. 3:**
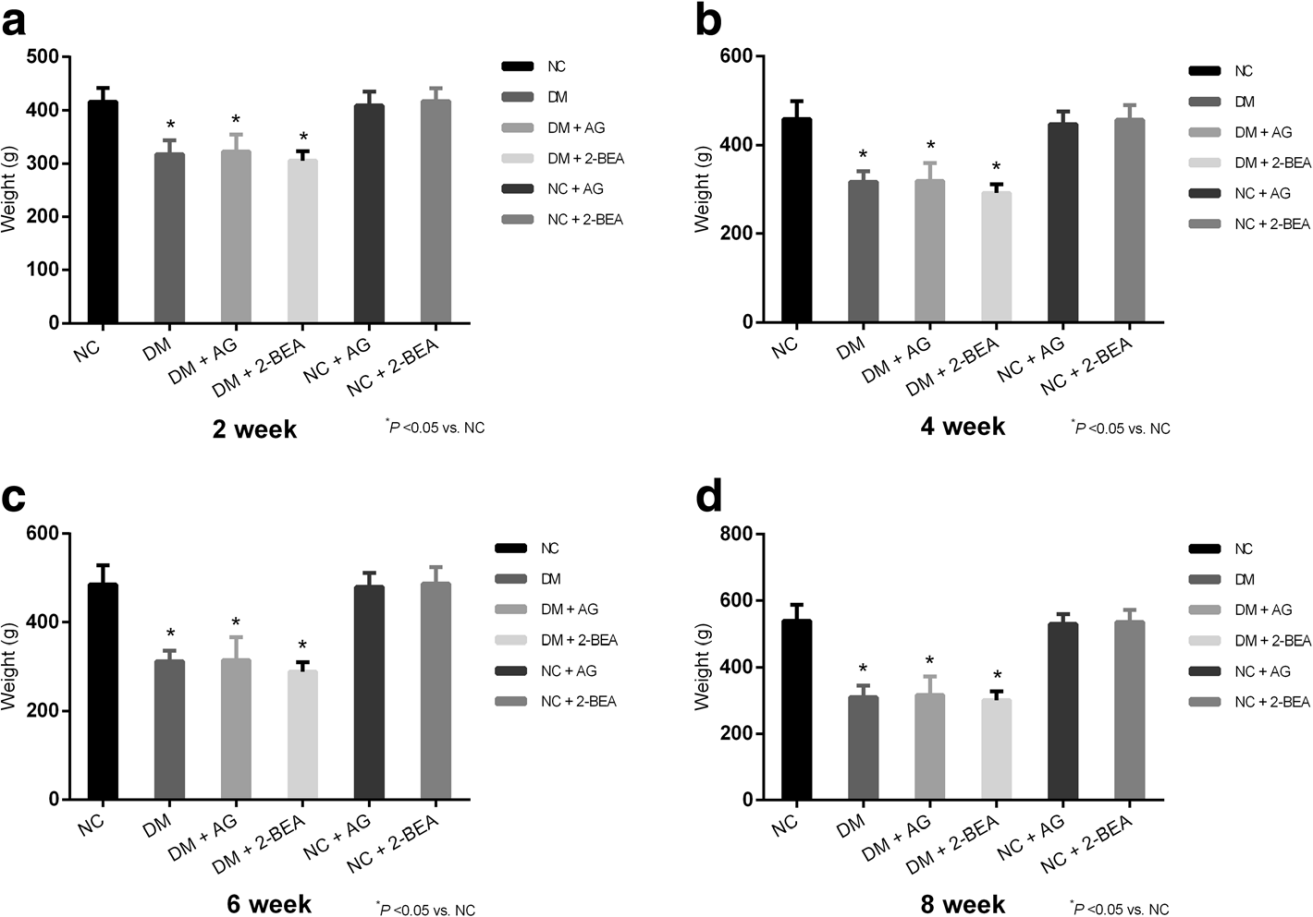
Comparison of body weight in different groups of rats (*n* = 10). After establishing the diabetic rat model, the body weight in the NC, DM, DM + AG, DM + 2-BEA, NC + AG, NC + 2-BEA groups was measured at respective time points of 2 (**a**), 4 (**b**), 6 (**c**), and 8 (**d**) weeks. Results are reported at the mean ± SD. ^*^P < 0.05 *νs.* NC group

The average blood glucose level of the rats in the NC group was 6.27 ± 0.85 mmol/L (range: 4.4–8.9 mmol/L). After establishing the diabetic rat model, the blood glucose levels among all the groups at respective time points of 2, 4, 6, and 8 weeks post-treatment are shown (Fig. 2a, b, c, d). When compared with the NC group, the blood glucose and body weight levels in the NC + AG and NC + 2-BEA groups were not significantly different, the blood glucose levels in the DM, DM + AG and DM + 2-BEA groups were significantly increased (*P* < 0.05). A significant reduction in the blood glucose level was also observed in the DM + 2-BEA group in comparison with the DM and DM + AG groups (*P* < 0.05). The body weight in the DM, DM + AG and DM + 2-BEA groups gradually rose or remained at a relatively stable level at each time point, which was significantly lower than that found in the NC group (*P* < 0.05) (Fig. 3a, b, c, d).

### Comparison of the SSAO activity in plasma and aorta

**Fig. 4:**
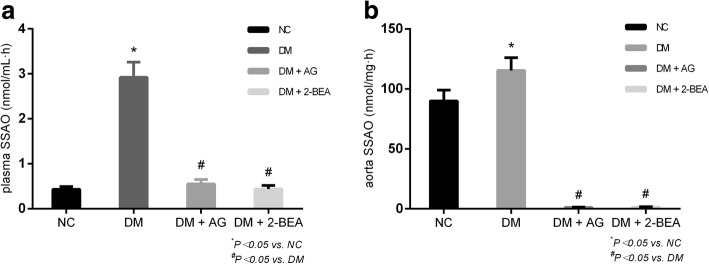
Comparison of SSAO activity in the plasma and aorta from different groups of rats (n = 10). Following AG (25 mg/kg) and 2-BEA (20 mg/kg for) therapy for 8 weeks in the rats, we measured the plasma SSAO (**a**) and aorta SSAO (**b**) by HPLC method in the NC, DM, DM + AG, DM + 2-BEA, NC + AG, NC + 2-BEA groups. Results are reported at the mean ± SD. ^*^ *P* < 0.05 *νs.* NC group, ^#^*P* < 0.05 vs. DM group

The SSAO activity in the plasma and aorta was significantly higher in the DM group than in the NC group (*P* < 0.05). Compared with the DM group, both the DM + AG and DM + 2-BEA groups showed obviously lower SSAO activity in the plasma and aorta (*P* < 0.05). However, the differences between the DM + AG and DM + 2-BEA groups were not significantly different (Fig. 4a, b).

### Comparison of the plasma levels of MA and FA

**Fig. 5:**
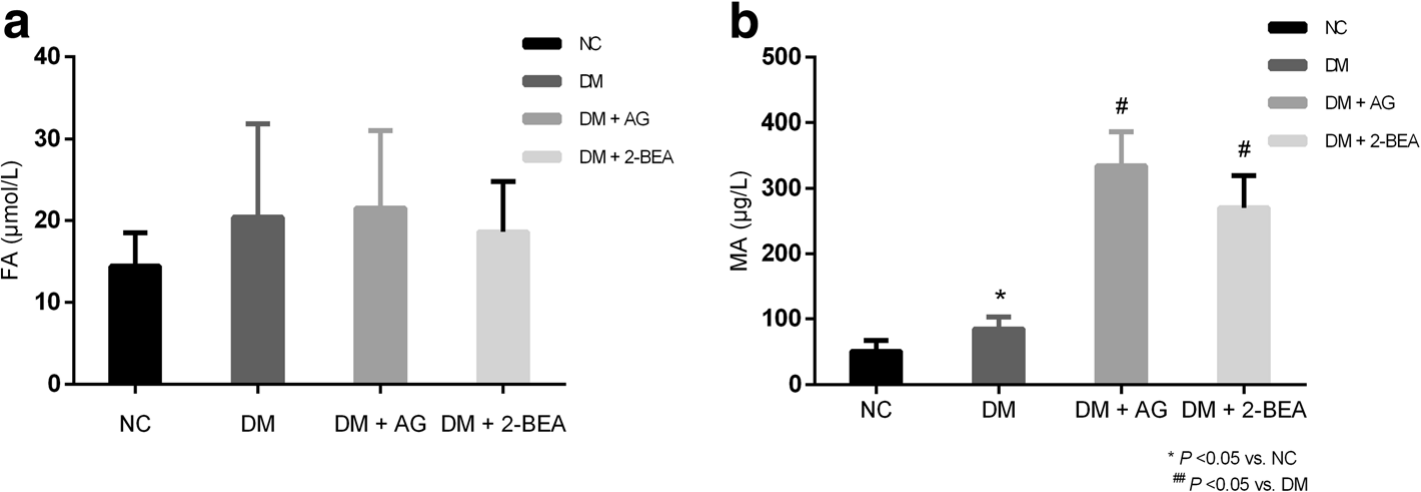
Comparison of plasma levels of FA anfd MA in different groups of rats (n = 10). AG and 2-BEA were SSAO inhibitors, upon AG (25 mg/kg) and 2-BEA (20 mg/kg) treatment for 8 weeks, plasma FA (**a**) and MA (**b**) levels which were the productions of SSAO-mediated oxidative deamination were measured. FA, formaldehyde; MA, methylamine; Results are reported at the mean ± SD. ^*^ *P* < 0.05 *νs.* NC group, ^#^ *P* < 0.05 vs. DM group

There were no significant differences in terms of plasma FA level among all the groups (Fig. 5a). Compared with the NC group, the DM group showed a significantly higher level of MA in the plasma (*P* < 0.05). Additionally, after treatment with AG and 2-BEA, the plasma MA level in the DM + AG and DM + 2-BEA group were obviously higher than that of the DM group (*P* < 0.05), but the differences between the DM + AG and DM + 2-BEA groups were not significantly different (Fig. 5b).

### Comparison of the plasma levels of NO(x)- and ET-1

**Fig. 6:**
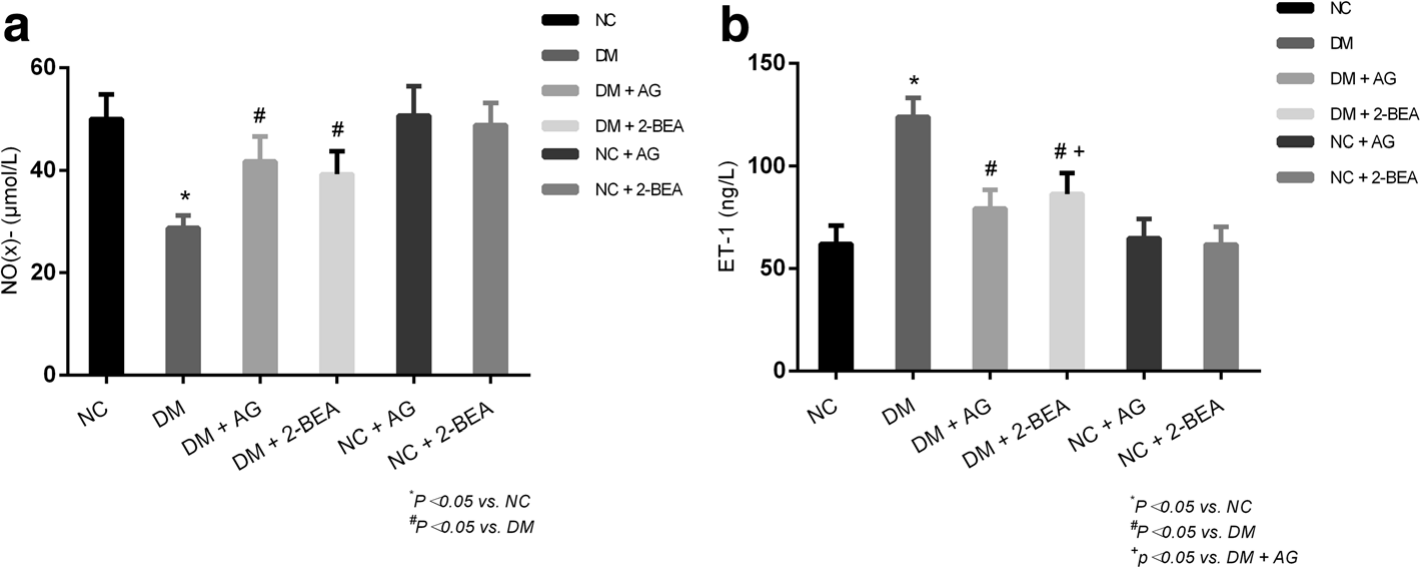
Comparison of plasma levels of ET-1 and NO(x)- in different groups of rats (n = 10). At the 8 weekends, NO(x)- was measured among all the groups (**a**), ET-1 was measured among all the groups (**b**). ET-1, endothelin-1; NO(x)-, nitrate/nitrite; Results are reported at the mean ± SD. ^*^ *P* < 0.05 *νs.* NC group, ^#^*P* < 0.05 vs. DM group, ^+^ *P* < 0.05 vs. DM *+* AG group

The plasma NO(x)- level was significantly reduced in the DM group compared with NC group (*P* < 0.05). Additionally, the plasma NO(x)- level was significantly higher in the DM + AG and DM + 2-BEA groups than in the DM group (*P* < 0.05), but there were no significant differences between the DM + AG and DM + 2-BEA groups (Fig. 6a). In addition, the plasma ET-1 level was significantly increased in the DM group in comparison with the NC group (*P* < 0.05). When compared with the DM group, a significant lower ET-1 level in the plasma was observed in the DM + AG and DM + 2-BEA groups (*P* < 0.05); however, differences between the DM + AG and DM + 2-BEA groups were significant (Fig. 6b).

### Morphological assessment of aorta

**Fig. 7:**
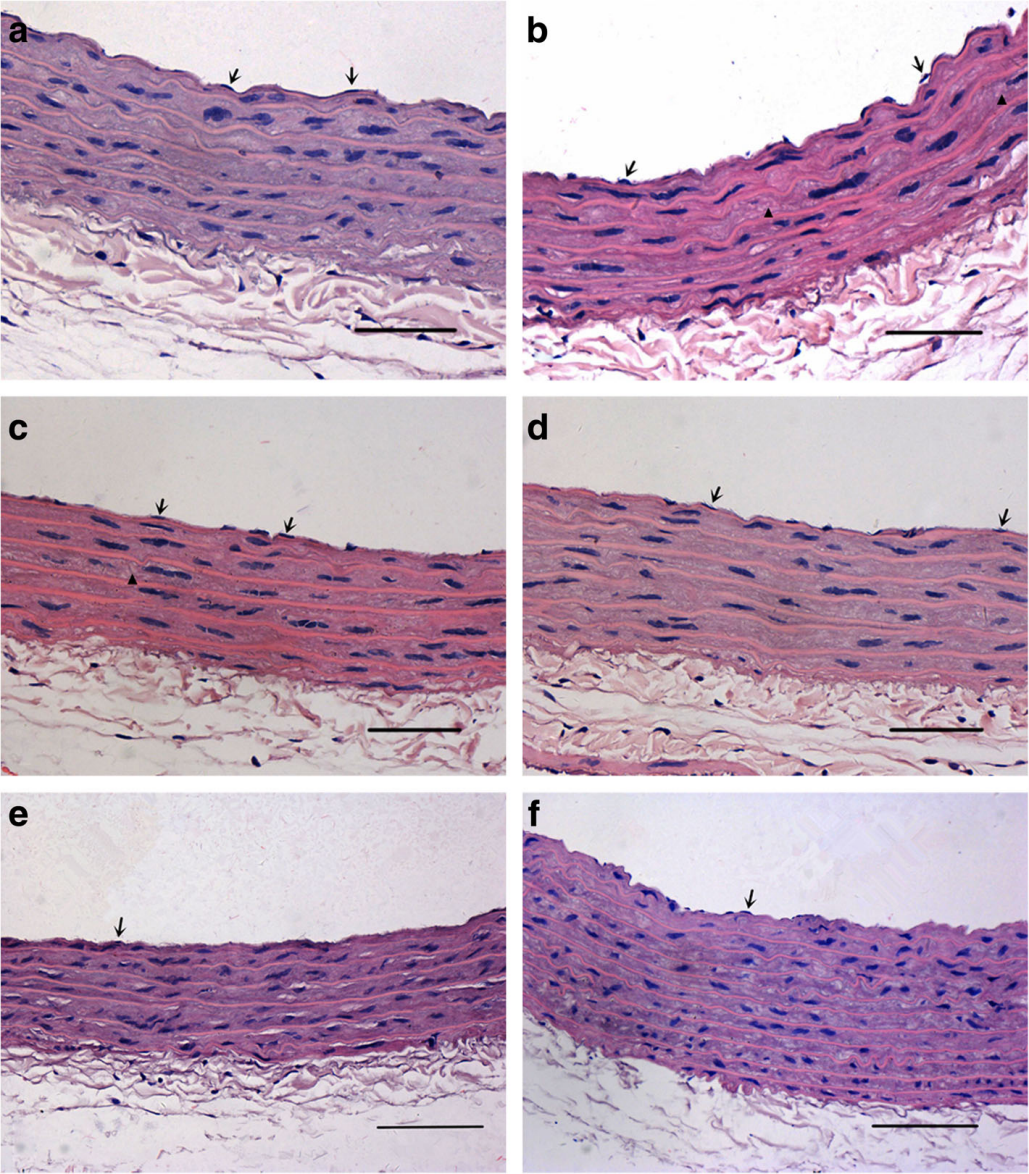
Morphological changes of aorta in different groups of rats by optical microscopy (H&E staining, × 400). **a**, NC group; **b**, DM group; **c**, DM + AG group; **d**, DM + 2-BEA group; **e**, NC + AG group; **f**, NC + 2-BEA group; Scale bar, 50 um; ↑, endothelium; ▲, matrix fiber

The aortic endothelial cells (ECs) of rats from the NC, NC + AG, NC + 2-BEA groups remained firmly attached to the internal elastic lamina, with no evidence of thickened middle elastic lamina (Fig. 7a, e, f). By contrast, rats from the DM group presented a marked swelling of the ECs under an optical microscopy with an isolated internal elastic lamina. Moreover, the matrix fiber and smooth muscle cells (SMCs) of the middle elastic lamina showed irregular arrangements, accompanied by obvious proliferative activity (Fig. 7b). In the DM + AG group, the aortic ECs were also closely attached to the internal elastic lamina. No evidences of swelling or detachment of ECs, or proliferation of SMCs and matrix fiber in the middle elastic lamina were detected (Fig. 7c). In addition, there was also a close attachment of aortic ECs to the internal elastic lamina in the DM + 2-BEA group, along with a regular arrangement of the matrix fiber and SMCs in the middle elastic lamina. No apparently-thickened middle elastic lamina or proliferation of SMCs and matrix fiber was detected (Fig. 7d).

**Fig. 8:**
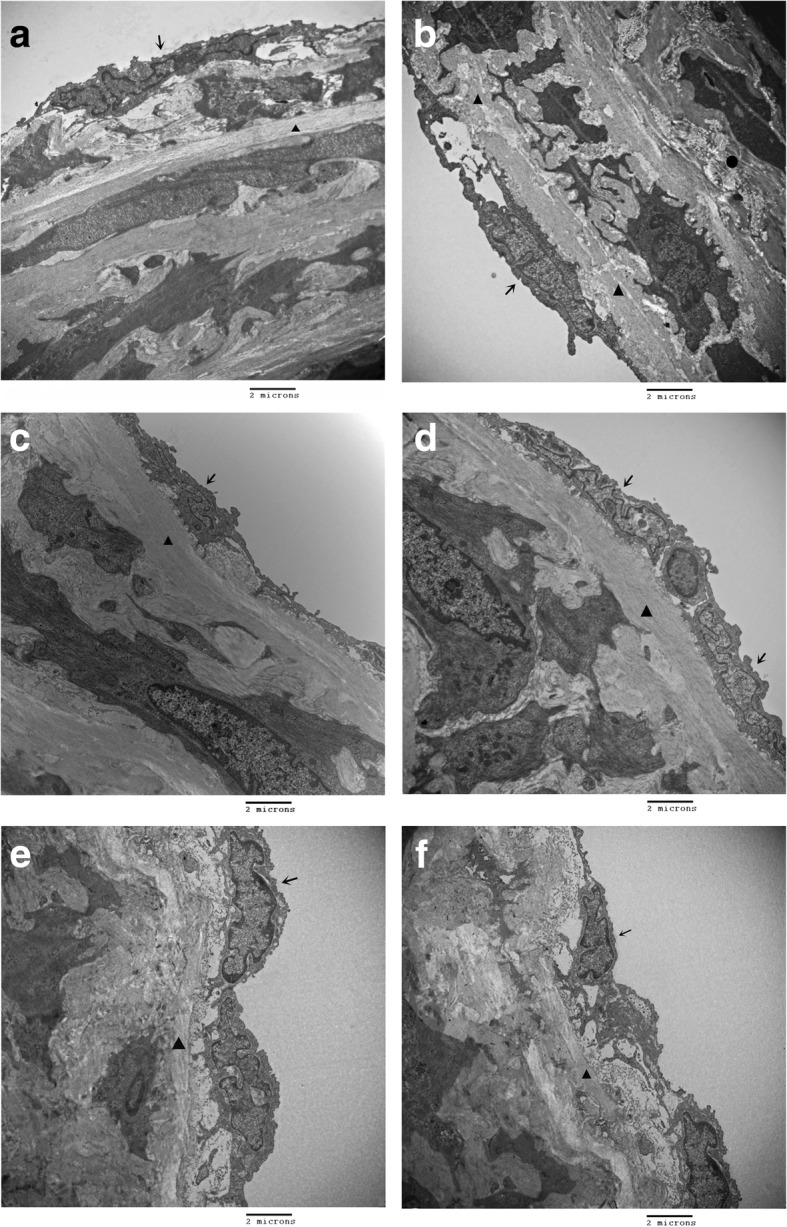
Morphological changes of aorta in different groups of rats by electron microscopy. **a**, NC group; **b**, DM group; **c**, DM + AG group; **d**, DM + 2-BEA group; **e**, NC + AG group; **f**, NC + 2-BEA group; Scale bar, 2 μm; ↑, endothelium; ▲, mitochondria; ●, matrix fiber

The aortic ECs of rats from the NC, NC + AG, NC + 2-BEA groups were flattened with little interstitial matrix under an electron microscopy, and the internal elastic lamina appeared to be straight and have a uniform thickness (Fig. 8a, e, f). Rats from the DM group presented swollen aortic ECs, and the internal elastic lamina became widened, accompanied by a non-uniform thickness and even rupture of the membrane. Moreover, an obvious proliferation of the matrix fiber and SMCs in the middle elastic lamina could be seen (Fig. 8b). Besides, the aortic ECs of rats from the DM + AG group, linked by tight junctions, exhibited a typical flattened morphology, along with the uniform internal elastic lamina. Nevertheless, the proliferation of SMCs and matrix fiber was not identified in the middle elastic lamina (Fig. 8c). In the DM + 2-BEA group, the aortic ECs were tightly adherent to the internal elastic lamina, and demonstrated tight junctions and a flattened morphology by electron microscopy. The H&E staining revealed a uniform thickness of the internal elastic lamina, with no abnormalities in the middle elastic lamina (Fig. 8d).

### Morphological assessment of kidney

**Fig. 9:**
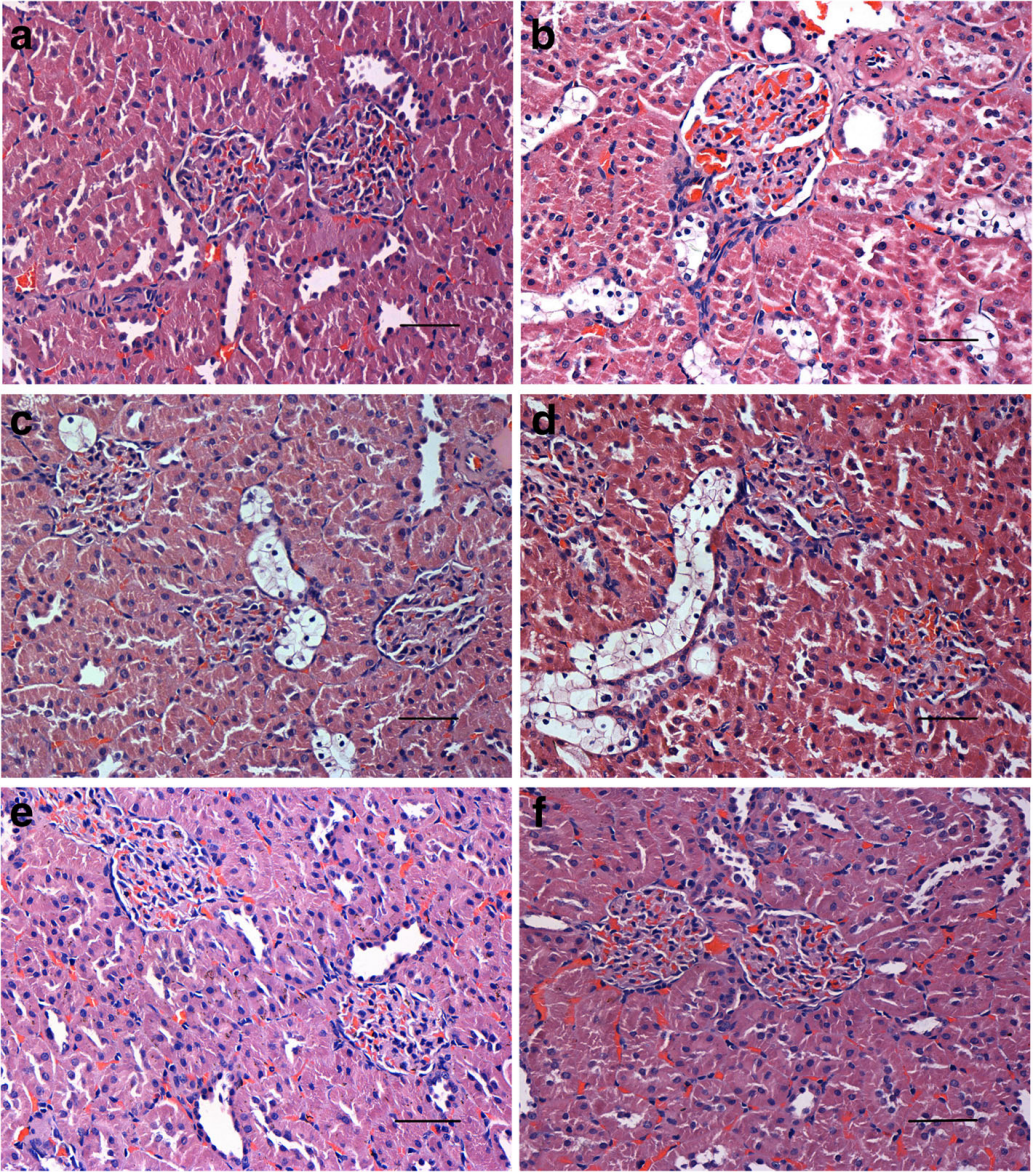
Morphological changes of kidney in different groups of rats by optical microscopy (H&E staining, × 400). **a**, NC group; **b**, DM group; **c**, DM + AG group; **d**, DM + 2-BEA group; **e**, NC + AG group; **f**, NC + 2-BEA group. Scale bar, 50 μm

Rats from the NC, NC + AG, NC + 2-BEA groups presented a normal glomerular shape under an optical microscopy. No evidences of glomerular capillary expansion and hyaline degeneration in the renal tubular epithelial cells were observed (Fig. 9a, e, f). By contrast, the H&E staining revealed enlarged glomeruli and apparent hyaline degeneration of renal tubular epithelial cells in the DM group (Fig. 9b). The enlargement of glomeruli could also be found in the DM + AG group with a slightly milder degeneration of renal tubular epithelial cells compared with the DM group (Fig. 9c). Along with the enlargement of glomeruli, a milder degeneration of renal tubular epithelial cells was noticed in the DM + 2-BEA group in comparison with the DM group (Fig. 9d).

**Fig. 10:**
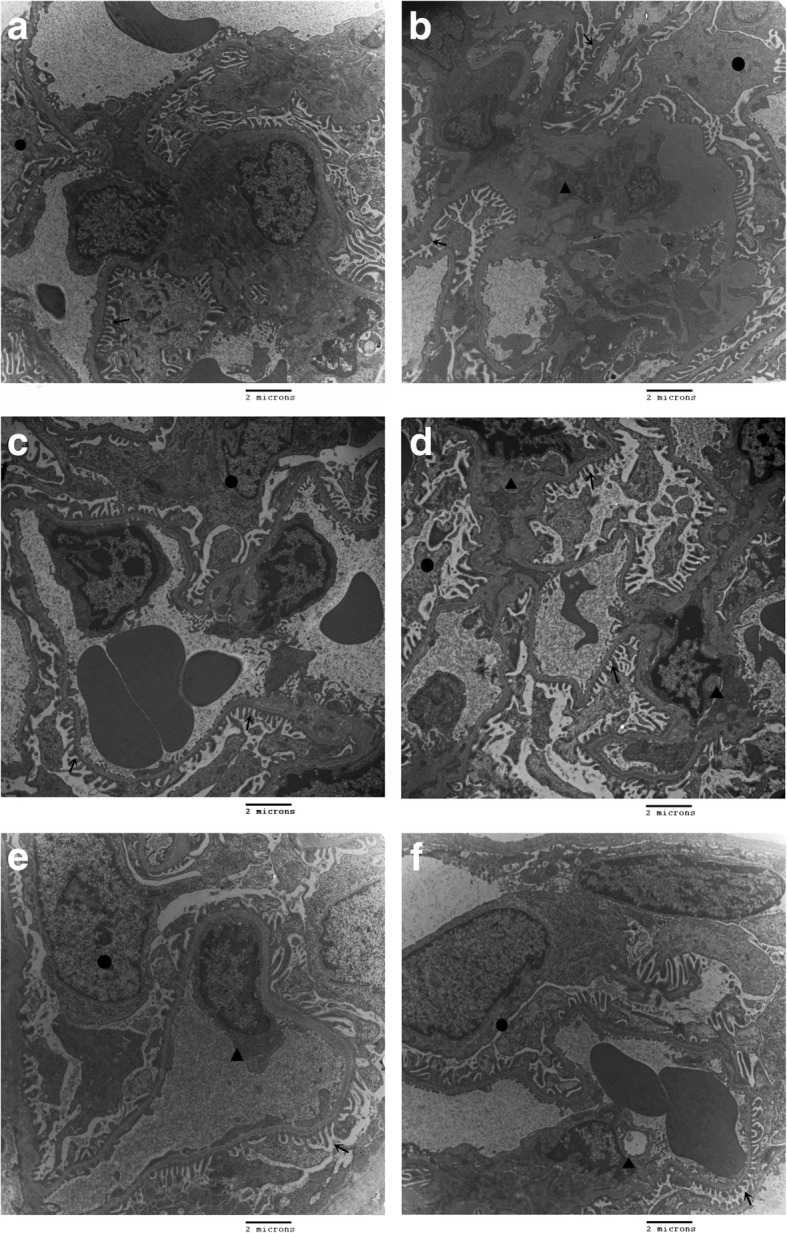
Morphological changes of kidney in different groups of rats by electron microscopy. **a**, NC group; **b**, DM group; **c**, DM + AG group; **d**, DM + 2-BEA group; **e**, NC + AG group; **f**, NC + 2-BEA group; Scale bar, 2 μm; ↑, podocyte foot processes; ●, podocyte; ▲, mesangial area

The electron microscopy showed a uniform thickness of glomerular basement membrane and regular arrangements of podocytes in the NC, NC + AG, NC + 2-BEA groups. No proliferation of mesangial cells and matrix was detected (Fig. 10a, e, f). In the DM group, glomerular basement membrane became thickened, along with irregular arrangements or fusion of podocytes. Moreover, obvious proliferation of mesangial cells and matrix could be seen (Fig. 10b). By contrast, no apparently-thickened glomerular basement membrane was detected in the DM + AG group. Moreover, regularly arranged podocytes and mild proliferation of mesangial matrix could be seen with little evidence of podocyte fusion (Fig. 10c). Rats from the DM + 2-BEA group also presented a regular arrangement of podocytes. However, no evidences of thickened glomerular basement membrane and proliferation of mesangial cells and matrix were observed (Fig. 10d).

## Discussion

The dynamic changes in the blood glucose levels and body weight were detected after establishing the diabetic rat model. The results showed that 2-BEA and AG have not prevented the changes in the blood glucose which has an implication in the vascular dysfunction. The T1DM model established in our study was successful and easily performed, along with high stability. Morphology changes of aorta and kidney can occur early in the T1DM model. In our experiment, the rat aorta was used to study the morphological changes of diabetic macrovessels, mainly due to the ease of collecting thoracic aorta, and the aortic atherosclerosis changes in the early stage of the model.

AG, a nucleophilic hydrazine, has been shown to be capable of blocking the formation of advanced glycation end products (AGEs) by binding to the intermediate products and their derivatives in the process for non-enzymatic saccharification. AG also exert an inhibitory effect on SSAO by irreversibly binding to the coenzyme 6-hydroxydopamine oxime residue of SSAO. Yu PH et al. reported that the SSAO activity of rat aorta can be irreversibly inhibited by AG in vitro. Moreover, the inhibitory effect of AG on aortic SSAO activity was found to be dose-dependent in STZ-induced diabetic rats following AG administration [9]. As a highly potent and selective inhibitor for SSAO, 2-BEA was used in this study as a positive control, and we evaluated the effects of AG and 2-BEA on the SSAO activity both in vivo and in vitro. The results showed that both AG and 2-BEA can result in a significantly reduced SSAO activity in vitro, and the inhibitory effect of AG seemed weaker when compared with that of 2-BEA. However, the long-term treatment strategy with AG in diabetic rats possessed similar inhibition of plasma SSAO activity as was shown in 2-BEA. Thus, AG was included as a potent SSAO inhibitor in the present study.

In mammals, two forms of SSAO have been identified, which are a tissue-bound form and a soluble plasma form. The tissue-bond SSAO activity is widely distributed in different tissues, particularly in vascular smooth muscle, retina, and adipose. The results from the present study revealed that compared with the control group, the SSAO activity in the aorta and plasma was significantly higher in the DM group, while the SSAO activity in the plasma of rats from the DM group was obviously lower in comparison with that in the aorta. This is probably because plasma SSAO mainly originates from extracellular digestion of tissue-bound SSAO and the release of SSAO from the injured tissues with high SSAO activity into the bloodstream; thus; the plasma SSAO appears to have similar biological properties to those of tissue-bond form [10]. It has been reported that SSAO mainly catalyzes the deamination of endogenous MA and aminoacetone to produce toxic FA, H_2_O_2_ and ammonia. FA has been shown to possess strong biological properties, and it can promote the release of methylol groups, Schiff-bases, and methylene bridges by reacting with monoamines and amides, which then contributes to the formation of irreversible cross-linked complexes between proteins, as well as between proteins and single-stranded DNA, thereby exerting its influence on the structure and function of proteins and DNA expression associated with endothelial damage [11]. As one of the end products of SSAO-mediated oxidative deamination, the H_2_O_2_ can be converted to toxic hydroxyl radicals via Fenton reaction. Meanwhile, the interactions between FA and H_2_O_2_ can further cause damage to vascular ECs [12]. In the current study, the SSAO activity was significantly elevated in the plasma and aorta of rats from the DM group. This observation was likely brought about by up-regulation in SSAO due to the increase in SSAO substrates like MA and toxic products from excessive SSAO-mediated oxidative deamination. Furthermore, SSAO is widely distributed in the vulnerable regions of diabetic vascular dysfunction, suggesting that SSAO could be closely associated with the occurrence and development of diabetic vascular dysfunction. We therefore speculated that the inhibition of SSAO-mediated oxidative deamination might be of great benefits for the treatment of vascular dysfunction of DM.

In the present study, an increase in the SSAO activity and plasma MA level was observed in the DM model group, while the plasma FA maintained a stable level. Based on these results, it was speculated that both AG and 2-BEA might be incapable of reducing the plasma FA level, although they can inhibit the SSAO-catalyzed oxidative deamination. There were a number of reasons for the stable level of FA in the rats from the DM model group. On the one hand, there are numerous sources of endogenous FA, including lipid peroxidation and histone demethylation [13], in addition to the SSAO-mediated deamination of MA. On the other hand, FA can not only be excreted in the urine by the formation of uric acid via enzymatic reaction, it can also be converted into carbon dioxide and breathed out of the body. Moreover, the non-enzymatic conversion of FA can cause protein dysfunction and misfolding as well as abnormal expression of DNA. Kazachkov M et al. have analyzed the radioactive residual activities in different tissues of mice. In their study, radioactively labeled [14C]-methylamine was administered via tail vein intravenous injection after pretreatment with AG, and the long-lasting radioactive protein residual activities were detected in all tissues, which suggested the production of FA due to SSAO-mediated deamination of MA [14]. The observations from this study indicated that the SSAO activity in the plasma and aorta was significantly lower in both the DM + AG and DM + 2-BEA groups than that found in the DM group, whereas the plasma level of MA in the DM + AG and DM + 2-BEA groups was obviously higher than that of the DM model group, indicating that both AG and 2-BEA can reduce the production of toxic FA and H2O2 by a significant inhibition of the SSAO-catalyzed oxidative deamination.

ET and NO(x)- are reported to be important vascular contraction and relaxation factors secreted by ECs, and their dynamic balance has great benefits in maintaining the normal vascular physiology, so they are considered as markers of vascular dysfunction. However, the hyperglycemia-induced damage to vascular ECs can cause an imbalance between the ET and NO(x)- system, suggesting a central role of ET and NO(x)- system in the pathogenesis of diabetic vascular dysfunction [15]. In the current study, a significant reduction in the plasma NO(x)- level was observed in the DM group, while the plasma ET-1 level was significantly increased in comparison with the NC group. These findings revealed that diabetic rats presented a certain degree of vascular dysfunction and both AG and 2-BEA can regulate the dynamic balance between ET and NO(x)- system, thereby improving endothelium-dependent vasodilatation. The IC50 of AG to inhibit SSAO is lower than to inhibit NOS, many studies have shown that the dose for AG to inhibit NOS is mostly 100 mg/kg/d. In this study, 25 mg/kg/d AG was intraperitoneally injected, showing no significant inhibition of NOS [16]. After eight weeks of treatment, rats in the DM group showed an elevated SSAO activity, along with vascular morphological changes in the aorta and kidney during the early stages of DM. AG and 2-BEA inhibit SSAO oxidative deamination to improve the morphological changes of aorta and kidney in diabetic rats, thereby preventing the occurrence and development of diabetic vascular dysfunction.

## Conclusion

In summary, AG was a common SSAO inhibitor with low toxicity. AG was selected in this present study and its role in the prevention of diabetic vascular dysfunction was explained in a new mechanism. We also found that AG and 2-BEA might exert their effects by reducing the production of toxic FA and H_2_O_2_ by a significant inhibition of the SSAO-catalyzed oxidative deamination. AG and 2-BEA might also be greatly beneficial in the morphological changes of aorta and kidney in diabetic rats.

## Data Availability

We are currently analyzing the data from a different perspective and planning a related study. Therefore, the data and material are not shared in the current state. However, the datasets used and/or analyzed during the current study are available from the corresponding author on reasonable request.

## Abbreviations

AGEs: Advanced glycated end products
ET-1: Endothelin-1
HPLC: High performance liquid chromatography
NO(x)-: Nitrate/Nitrite
SSAO: Semicarbazide-sensitive amine oxidase
